# Analysis of clothing pressure based on material of virtually-fitted swimsuits and wearer body type and movement using 3D fashion design software

**DOI:** 10.1371/journal.pone.0334782

**Published:** 2025-11-05

**Authors:** Jisoo Kim, Youngjoo Chae

**Affiliations:** Department of Clothing & Textiles, Chungbuk National University, Cheongju, Korea; St Mary’s University, UNITED STATES OF AMERICA

## Abstract

This study aimed to evaluate and compare the clothing pressure of adult women’s full-body swimsuits based on body type, swimming posture, and fabric material. By using the CLO 3D virtual fitting system, we conducted both qualitative and quantitative analyses to assess pressure distribution across four major body regions during five swim-related movements. Five female body types—hourglass, apple, rectangle, triangle, and inverted triangle—and five movements—overhead arm raise, forward bend, T-pose, flutter kick, and cobra pose—were analyzed. The swimsuits used two fabric types—nylon 80% + spandex 20% blend fabric and polyester 80% + spandex 20% blend fabric; garment pressur**e** was measured at the chest, back, waist, and abdomen. The findings showed that CLO 3D had limitations in precisely distinguishing the characteristics of nylon and polyester, specifically in terms of clothing pressure and overall comfort. Significant variations in clothing pressure across different body parts were observed depending on swimming movement and body type. According to body type, the highest pressure was on the abdomen for the apple shape and the chest for inverted triangle shape. Regarding movement, the highest pressure was on the abdomen during the forward bend and chest during the cobra pose, indicating possible discomfort in those areas. Moreover, while the CLO 3D system effectively analyzes garment pressure in relatively static conditions of the wearer, it has limitations in assessing underwater environments and wearers’ dynamic states. This study recommends configuring the underwater environment, establishing a database of swimsuit fabric properties in a wet state, incorporating movements, and automatic correction of discrepancies between measured compressive force data and CLO 3D output. The findings provide a data-driven design direction to enhance wearability and functionality in swimsuit development, while presenting new possibilities for CLO 3D-based sportswear research without the need to use traditional human-wear trials.

## 1. Introduction

Swimming is considered a major event in international sporting competitions, such as the Olympics, and is widely enjoyed both as a competitive sport and recreational activity. Since it can be performed both indoors and outdoors, swimming remains accessible to people of all ages, regardless of season and weather conditions. Although the swimwear industry witnessed a temporary downturn following reduced indoor pool usage during the pandemic, it is now showing signs of recovery and a positive outlook after the reopening of swimming facilities, increased demand for overseas travel, and a steady rise in the athleisure trend. Swimsuits are continuously evolving into high-functionality products, incorporating elements such as athletic functionality, hydrodynamics, body protection, and durability, transcending beyond basic everyday wear.

Swimsuits can be categorized into performance swimsuits, which emphasize functionality for sports competitions, and fashion swimsuits, which prioritize aesthetics for leisure activities, such as resort wear. In particular, the material and design in performance swimsuits have been widely reported to have a significant impact on reducing race times [1]. Accordingly, there is increasing emphasis on the need for research focusing on enhancing swimsuit performance. Furthermore, swimsuits incorporate various high-performance materials, including spandex, nylon, and polyester, to optimize properties such as stretchability, clothing pressure, and resistance reduction. Clothing pressure—defined as the force exerted by a garment on the surface of the human body—is a key factor that influences both swimming efficiency and overall comfort. As such, swimsuits are garments where functionality takes precedence over aesthetics, making the analysis of garment pressure, a key factor for both swimming efficiency and overall comfort, a critical factor in product development.

Recent research on swimsuits can be broadly categorized into three groups: swimsuit preferences, properties of swimsuit materials, and swimsuit design and pattern development. Regarding swimsuit preferences, Ueno [2] found that rash guards help alleviate anxiety and the psychological burden associated with body exposure, fostering a more positive attitude toward swimming classes. Li and Leonas [3] discovered that female Generation Z consumers, typically defined as individuals born between 1997 and 2012, tend to consider environmental impact and brand sustainability when selecting swimsuits, while design and functionality also play key roles influencing their decision-making. Pedro and Sandes [4] examined the similarities and differences in consumers’ swimwear choices between Brazil and Israel. They found that while Israeli respondents displayed a more open preference for designs from diverse cultural backgrounds, Brazilians tended to prefer designs from their culture. Meanwhile, Jiang et al. [5] analyzed factors influencing swimsuit purchasing decisions among 3,348 Chinese women, and determined that body fit was the most important consideration, regardless of whether it was a one-piece or two-piece swimsuit; two-piece swimsuits were the most preferred type in terms of wearer comfort.

With regard to the properties of swimsuit materials, Čubrić, et al. [6] revealed that environmental factors in swimming pools, such as chlorine-treated water, ultraviolet (UV) rays, and temperature, can alter the physical properties of swimsuit materials, leading to performance degradation. Li et al. [7,8] studied the effects of inlaid material, yarn, and knitted structure on the net buoyant force and mechanical properties of inlaid knitted fabric for buoyant swimwear. The findings showed that the net buoyant force and mechanical properties of the fabric are significantly affected by inlaid material and knitted structure, but not by the yarn. Kocak [9] examined swimwear fabric made of 100% polyester and 50% polyester–50% recycled polyester fibers in terms of their performance properties, including color fastness, abrasion resistance, and seam strength. Encan [10] found that UV exposure significantly alters swimwear fabrics’ breaking strength and elongation properties, elasticity, stiffness, and air permeability.

Finally, regarding research on swimsuit design and pattern development, Li et al. [7,8] developed a buoyant swimming vest using inlay knitting technology and compared its performance with two commercially-available samples. They found that the developed vest received higher user satisfaction in terms of fit, comfort, and mobility. Hashish et al. [11] developed a one-piece flat pattern for printed beachwear cover-ups to minimize cut-and-sew waste and achieve sustainability. Afifi et al.’s [12] study on swimwear pattern-making using Adobe Illustrator, aligns with the recent digitalization of the fashion industry, while proposing a relevant curriculum. Li et al. [13] reviewed the significant advancements and opportunities in swimsuit design, predicting that smart textiles and technologies will be increasingly applied to swimsuits for purposes such as real-time monitoring and instruction, intelligent drag reduction, and energy harvesting.

Although various studies have investigated swimsuits, none has quantitatively analyzed surface pressure—a key factor affecting fit, comfort, and exercise efficiency. Prior research on clothing pressure has primarily focused on sportswear [14–16], compression garments [17–19], and medical wear [20–22], emphasizing functions such as thermoregulation, muscle stabilization, and fatigue recovery. However, swimsuits are functionally distinct in that they are designed for underwater use, where the body would typically experience hydrodynamic drag, buoyancy, and significant deformation. While the present study did not simulate underwater conditions, such environmental factors highlight the limitations of current digital simulation tools and underscore the need for specialized analysis when studying swimsuits. As such, although swimsuits share functional characteristics with compression wear, they require a more specific analysis under aquatic conditions. To date, no study has examined swimsuit pressure using a digital system to assess body type, pose, and material—moving beyond subjective evaluations.

CLO 3D, a digital fashion design system widely used in the fashion industry, is a three-dimensional design software that offers virtual true-to-life garment visualization. The product simulations minimize the need for unnecessary physical samples during the design process, while enhancing the efficiency of design-related communication between companies. In addition to reducing cost and shortening product planning and production timelines through the use of simulations or 3D virtual samples, improved workspace efficiency can also be anticipated. CLO 3D’s virtual fitting technology serves as an efficient evaluation tool, not only in terms of design but also performance, as it allows for the prediction and visualization of garment pressure on the wearer within a digital environment, eliminating the need for a physical product. Using the CLO 3D fabric simulation function, clothing pressure of swimsuits can be easily analyzed, both visually and quantitatively, across different body types, movements, and material conditions. This can address the temporal and economic limitations of existing research that employs traditional human wear trials. Liu et al. [23] reported that clothing pressure data from a 3D virtual fitting function showed high accuracy in predicting pant fit. However, their study was limited to lower garments, specifically pants, and involved relatively simple postures with a limited range of motion, such as sitting, walking, and raising arms. Therefore, it fails to comprehensively reflect the fitting characteristics and comfort of full-body sportswear/compression-wear products for various body types during different physical activities involving a wide range of body movements.

Accordingly, the present study used the CLO 3D virtual fitting system to analyze the surface pressure of full-body compression-wear, specifically adult women’s full-body swimsuits made from different materials, on different body parts, based on female body types and swimming movements. Moreover, the study verified whether it is possible to predict sufficient and accurate fit and wearer comfort of functional garments based solely on the data obtained from these virtual simulations without physical-sample creation. The analysis is expected to provide foundational data that can be used for designing and developing products in the swimwear industry. Furthermore, this study evaluated the material and fabric representation capabilities of a current 3D virtual fitting system and validated its garment pressure data through mannequin-based physical measurements with pressure sensors. Based on these findings, it proposes directions for the future development of the fashion industry, digital fashion education, and related research from a methodological perspective.

## 2. Methods

### 2.1. 3D virtual try-on garment creation

**Fig 1:**
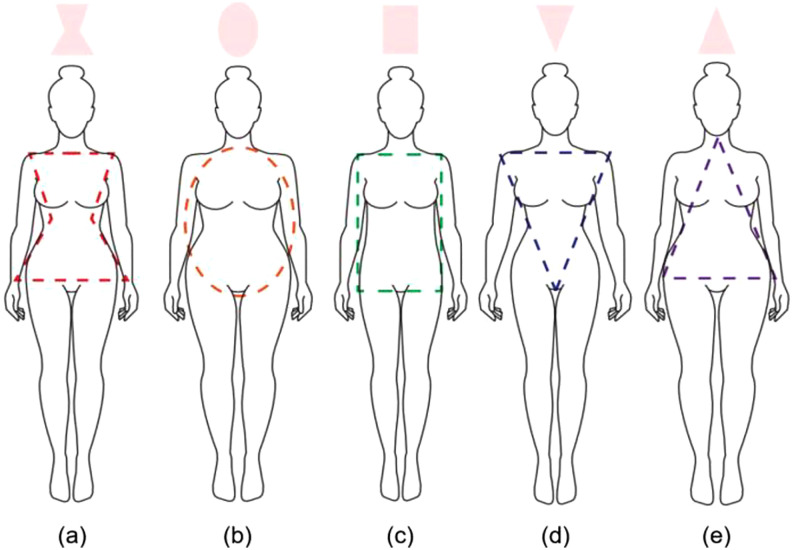
Five major female body types: (a) hourglass, (b) apple, (c) rectangle, (d) triangle, and (e) inverted triangle shapes.

Three-dimensional virtual try-on adult women’s full-body swimsuits were created using CLO 3D, based on a female one-piece swimsuit in medium (M) size from the French brand “Arena,” a globally-recognized name in the swimsuit market. The pattern was created with the following dimensions: 68 cm total length, 36 cm shoulder width, 90 cm bust circumference, 72 cm waist circumference, 22 cm armhole depth, 54 cm thigh circumference, and 40 cm of hem width (Table 1). Five female body types were selected based on the international standard size ISO 8559-1: hourglass, apple, rectangle, triangle, and inverted triangle shapes (Fig 1).

**Table 1:** The size of the swimsuit pattern (unit: cm).

Total length	Shoulder width	Bust circumference	Waist circumference	Armhole depth	Thigh circumference	Hem width
68	36	90	72	22	54	40

In the hourglass shape, the chest and hips are large and well-balanced, with a well-defined waist, while the apple shape is characterized by fat accumulation in the upper body, particularly around the abdomen and waist. The apple body type tends to have a larger chest and shoulders, a thicker waist, and a relatively slimmer lower body (hips and legs). The rectangle body shape has an overall straight silhouette, as there is little difference between the waist and hip area measurements. The chest, waist, and hip have similar widths, and this shape is often observed in slimmer body types. Moreover, it is often characterized by less prominent body curves owing to lower muscle mass. The triangle body shape features a narrow waist and a relatively smaller upper body, whereas the hips and thighs are more developed. This body type tends to have a wider hip area and fat concentrated in the lower body. Finally, the inverted-triangle shape has broad shoulders and well-developed chest, with relatively narrow waist and hips. This body type creates a silhouette where the upper body appears larger and the lower body looks slimmer [24]. The body measurements of the avatar in CLO 3D software were configured according to these five body types (Table 2). The 3D virtual try-on garment was developed through avatar creation, pattern input, sewing, and layout, culminating in a final simulation. During the virtual try-on, the relevant values are entered into the fabric property window of the software to implement its physical properties.

**Table 2:** Avatar sizes for five body types (unit: cm).

Body shape	Height	Shoulder width	Bust circumference	Waist circumference	Hip circumference	Thigh circumference	Torso length
Hourglass	165	37	94	70	98	56	150
Apple	165	38	96	78	94	55	150
Rectangle	165	36	88	72	92	53	150
Triangle	165	35	86	70	100	58	150
Inverted Triangle	165	40	98	74	92	52	150

**Fig 2:**
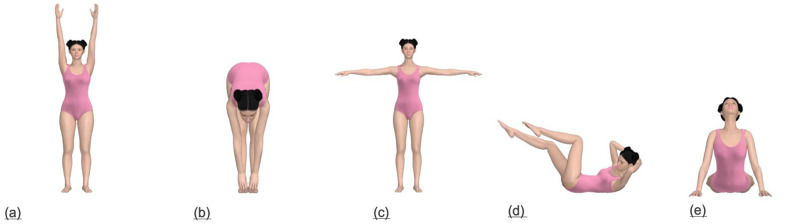
The five swimming poses examined in this study. (a) Overhead arm rise, (b) Forward bend, (c) T-pose, (d) Flutter kick position, and (e) Cobra pose.

This study selected five poses—overhead arm raise, forward bend, T-pose, flutter kick position, and cobra pose—to effectively analyze the garment pressure of female swimsuits (Fig 2). These poses closely represent actual swimming movements and are designed to assess key areas where changes in surface pressure occur during the simulation process. The poses were selected to include various joint flexion areas and a range of body movements. The first movement, the overhead arm raise, is similar to the preparatory position before starting swimming and was selected to analyze changes in compressive force in the shoulder straps, chest, armpit, and back areas of the swimsuit. The second movement, the forward bend, representing the position while preparing for diving, was used to analyze changes in clothing pressure in the waist, abdomen, and chest areas. The third movement, T-pose, a shoulder and upper-body stretching position, helps analyze changes in garment pressure in the chest and back areas. The fourth movement, the flutter kick position, reflecting the freestyle or backstroke kicking position, evaluates changes in clothing pressure in the abdomen, hip, and waist areas. Finally, the cobra pose, which is based on the freestyle stroke position, was chosen to analyze the changes in garment pressure in the chest, abdomen, waist, and back. The selection of these poses was informed by prior studies on aquatic motion patterns, body-fit considerations, and pressure measurement methodology for functional garments [1,5,27].

### 2.2. Clothing pressure measurement

**Fig 3:**
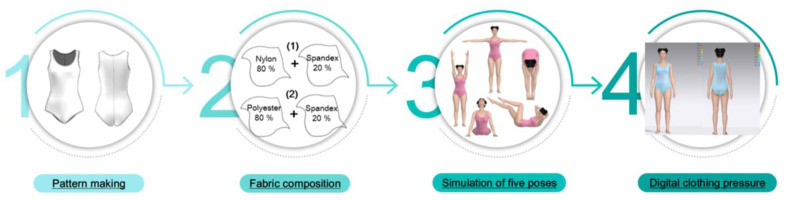
Schematic of the research process.

After the swimsuit patterns were created using CLO 3D, swimsuit material information was input. Female avatars with five distinct body shapes were assigned five poses each to generate virtual try-on samples, and clothing pressure measurements were recorded (Fig 3). Female swimsuits from the “Arena” brand used for this study are generally made with nylon/spandex or polyester/spandex blend fabric. Accordingly, the measurement was conducted for two types of fabrics: Fabric A (nylon-spandex blend, 80:20) and Fabric B (polyester-spandex blend, 80:20). By applying identical content of spandex, which has stretchability and compressive properties and is typically used in sportswear and compression-wear, the difference in surface pressure between polyester and nylon was observed for the two selected fabrics.

**Fig 4:**
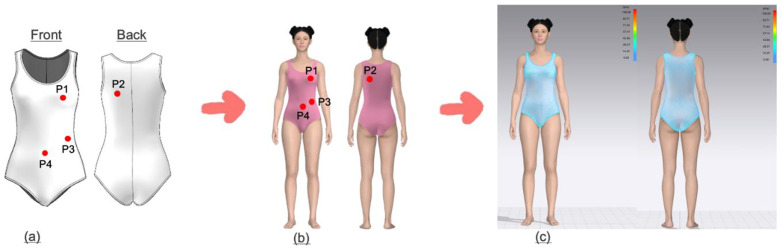
Digital clothing pressure measurement by virtual try-on. (a) Measuring points, (b) Virtual try-on, and (c) Measuring cloth pressure.

To measure clothing pressure, four body parts were selected based on their contact with the swimsuit, areas that affect wearability and movement during exercise, and areas that held relevance in previous studies on sportswear and compression-wear [25]: chest, back, waist, and abdomen (Fig 4a). The chest (P1) is the area where the shoulder straps and upper fabric experience the greatest tension when wearing the swimsuit, making it an important indicator for evaluating compressive force and fit stability. The back (P2) is where the tension and fabric pressure are most concentrated, making it essential to analyze the fabric’s balance and wearer comfort according to movement. The waist (P3) is the primary point where the swimsuit fits closely to the body and holds the body shape in place, with the level of pressure in this area significantly affecting overall wearability and mobility. The abdomen (P4) is where the fabric fits most closely to the body, making it is essential to analyze comfort and the fabric’s flexibility when experiencing pressure during physical activity.

Clothing pressure in the virtual fitting environment was analyzed using the stress distribution feature of the 3D view interface in CLO 3D. The pressure values for each of the four designated body areas were obtained by selecting specific points using the mouse cursor. Since the garment pressure measurements in this study were conducted in a virtual environment, the unit “kPa” hereafter refers to virtual kPa [26]. In CLO 3D, the compressive force intensity is visually represented through a color map. Blue indicates minimal contact between the garment and body, reflecting low pressure, while green-to-yellow represents moderate pressure, indicating a comfortable fit, and red reflects high pressure, indicating potential discomfort caused by excessive tightness.

### 2.3. Data analysis

The compressive force across the four body parts of the swimsuit were analyzed both qualitatively and quantitatively for five swimming poses. The qualitative analysis emphasizes the stress distribution displayed through the 3D view function of the software, represented by the color map. The stress color map was examined from the front, side, and back angles of the virtual garment sample, allowing for the observation of variations in stress colors—blue (low pressure), green-to-yellow (moderate pressure), and red (high pressure)—according to different swimming poses and fabric types.

For the quantitative analysis, clothing pressure values measured for each body part were numerically compared across five swimming poses and two fabric types. Based on the findings from both qualitative and quantitative analyses, this study proposes an optimized simulation function within 3D fashion design software, enhancing material selection and garment fit through accurate garment pressure assessment. To ensure reliability, each simulation condition was repeated three times, and the mean pressure values were used for analysis.

### 2.4. Mannequin-based wear trial

A supplementary mannequin-based experiment was conducted to assess whether the clothing pressure results obtained from the CLO 3D simulation reflected pressure distribution patterns comparable to those observed in physical measurements. Separately from the body-type comparison, this experiment used a physical dressing mannequin configured to represent the hourglass body type, which served as the reference shape in the virtual simulations. The same swimsuit prototypes (Fabric A: 80% nylon and 20% spandex; Fabric B: 80% polyester and 20% spandex) were fitted onto the mannequin under identical conditions.

The mannequin was manually positioned into three static postures that could be structurally supported: T-pose, forward bend, and overhead arm raise. Clothing pressure was measured in each posture. As the body shape remained constant, the analysis did not focus on absolute pressure differences between the two fabrics. Instead, it aimed to examine whether the pressure distribution tendencies across postures aligned with those obtained from the CLO 3D simulation.

An inflatable-type pressure sensor system (AMI3037102, AMI Techno, Japan) was used for measurement. All tests were conducted twice in a sealed indoor environment maintained at 23°C under controlled humidity conditions. Pressure values were collected from four anatomical regions: chest (P1), back (P2), waist (P3), and abdomen (P4). To compare the results of the mannequin-based measurement with the CLO 3D simulation results, a repeated-measures analysis of variance (ANOVA) was performed using SPSS 28.0 (IBM, USA). Posture (three levels) was treated as the within-subject factor, and measurement method (CLO vs. mannequin) as the between-subject factor.

This mannequin-based pressure evaluation approach was informed by the smart manikin system introduced by Wang and Zhang (2013) [27], as well as the comparative pressure analysis framework between mannequins and human subjects proposed by Liu et al. (2016) [28]. Prior studies have demonstrated that mannequin-based testing can serve as a reliable substitute for human wear trials, providing repeatable and quantitative data to complement virtual simulation outcomes.

## 3. Results and discussion

### 3.1. Qualitative analysis of the differences in clothing pressure according to materials, wearer body type, and movement

This study analyzed the differences in clothing pressure of adult women’s full-body swimsuits based on fabric material as well as body type and movements using CLO 3D. Accordingly, five swimming poses—overhead arm raise, forward bend, T-pose, flutter kick, and cobra pose—were selected for analysis, along with two fabric types: Fabric A (an 80:20 nylon–spandex blend) and Fabric B (an 80:20 polyester–spandex blend). Nylon has exceptional stretchability and flexibility; therefore, the surface pressure can vary significantly depending on body type and movement. However, polyester has lower stretchability and elastic recovery than nylon, resulting in a relatively higher compressive force and lower variation depending on body type and movement [29]. To assess whether the CLO 3D virtual fitting system effectively represents the clothing pressure based on different types of material, body types, and poses, the garment pressure was first analyzed qualitatively based on the color maps of stress distribution provided by the software.

**Fig 5:**
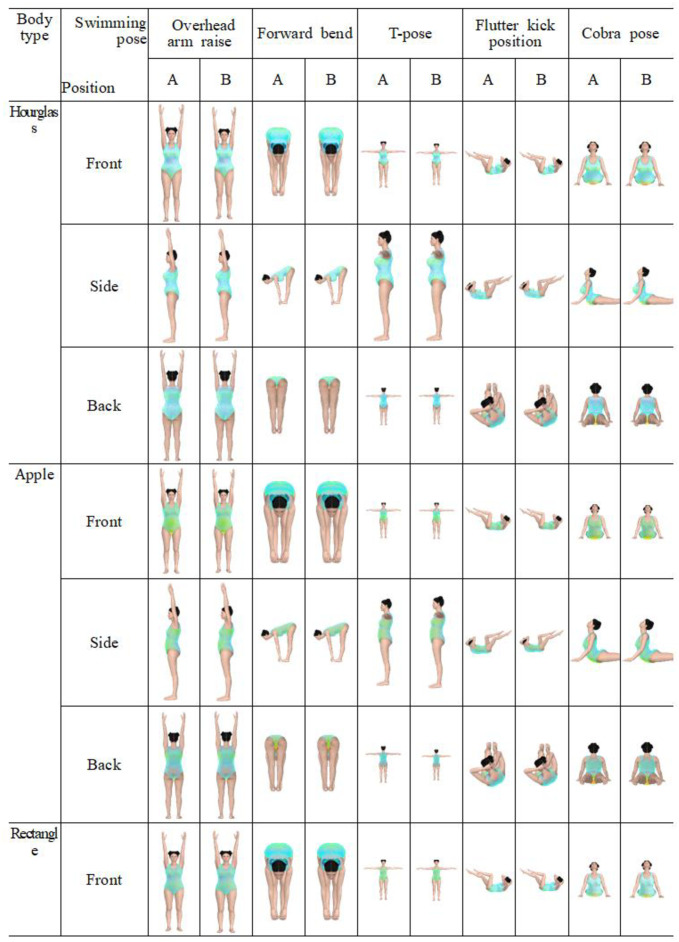
Garment Fit Maps for five swimming poses and two types of fabrics (A: Nylon 80% + Spandex 20%, Polyester 80% + Spandex 20%). Note. In each image, blue indicates lower garment pressure at the corresponding body part, green–yellow represents medium clothing pressure, and red reflects high compressive force.

Fig 5 presents the stress color map for the five swimming poses provided by the CLO 3D view function. As shown by the different colors displayed on the map, compressive force on each body part can be identified according to differences in material, body type, and movements. In general, during movements while wearing swimsuits, medium clothing pressure was observed in the chest and abdomen, indicated by green–yellow, whereas relatively lower surface pressure was observed in the back and waist, indicated by green–blue. The composition customization feature was upgraded in the latest version of CLO 3D (ver. 2024.2.120), allowing users to directly enter the fiber blending ratio of the material as a percentage (%). However, although the two fabric types selected in this study are composed of different fibers, it is difficult to qualitatively observe the differences in garment pressure through images A and B, as illustrated in Fig 5.

Depending on the body type of swimsuit wearers, the qualitative analysis revealed that apple and rectangle shapes exhibited more green–yellow compared to other body types, indicating generally higher clothing pressure. However, the findings from the qualitative analysis are contrary to the quantitative analysis, which will be discussed in the next section (mean clothing pressure obtained by quantitative analysis: hourglass 36.73 kPa; apple 35 kPa; rectangle 32.03 kPa; triangle 35.46 kPa; and inverted triangle 36.18 kPa). Moreover, the areas where garment pressure was higher or lower varied depending on the body type. For example, in the apple shape, a wide distribution of green–yellow colors was generally observed in the chest and abdomen regardless of movement, indicating relatively high surface pressure. However, the triangle shape appeared more bluish across a wider range of the body compared to other body types, indicating low garment pressure, contrary to the aforementioned quantitative analysis.

**Fig 6:**
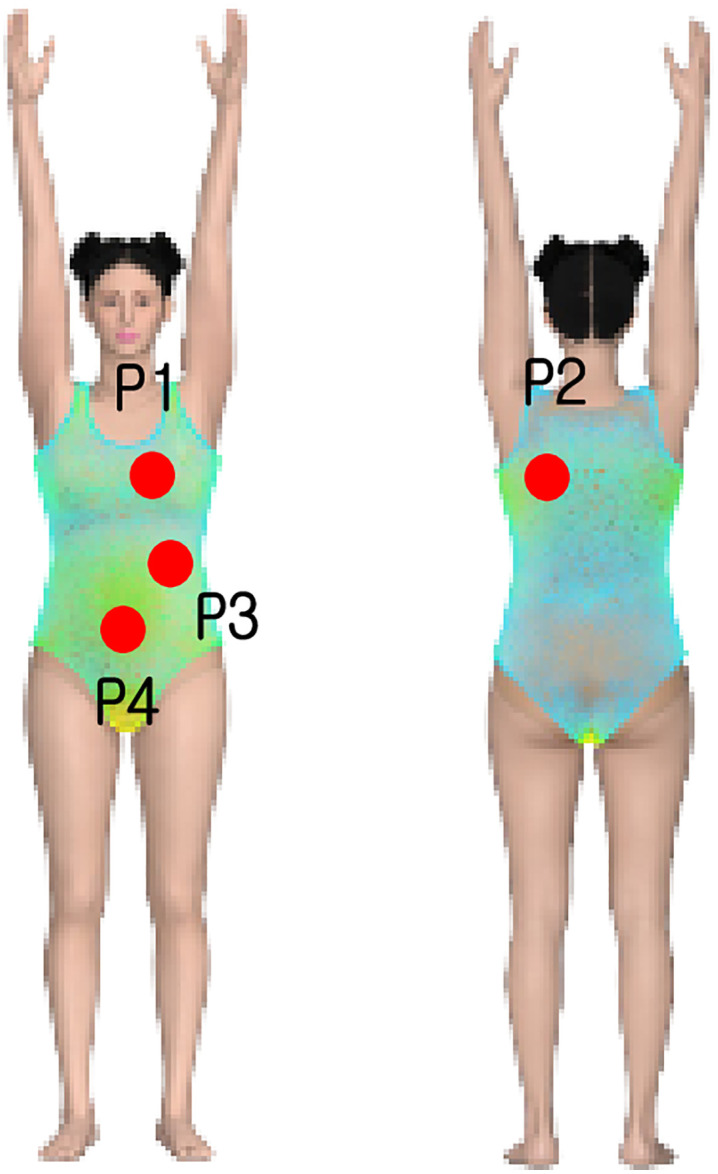
Enlarged front and back views with labeled pressure zones (Apple body type, overhead arm raise posture).

The images in Fig 5 show that there are differences in clothing pressure depending on the movement of the swimsuit wearer. However, because the stress color map alone does not allow for a specific and accurate identification of the presence and extent of surface pressure differences, it is necessary to conduct a quantitative analysis of compressive force based on swimsuit materials, body type, and movements. To support interpretation of local pressure distribution, Fig 6 presents enlarged front and back views of the Apple body type in the overhead arm raise posture. Labeled regions (P1–P4) indicate high-pressure areas, with the visualization aligned with predefined measurement points described in Fig 4.

### 3.2. Quantitative analysis of the differences in clothing pressure according to material, body type, and movements

An earlier analysis of the CLO 3D stress color map revealed no significant difference in surface pressure between the nylon-based fabric (nylon 80% + spandex 20%: A) and polyester-based fabric (polyester 80% + spandex 20%: B). Moreover, it is not possible to specifically and accurately identify the presence and extent of clothing pressure differences based on body type and movement. Accordingly, a quantitative analysis was conducted based on garment pressure values obtained using the same CLO 3D virtual fitting system. Table 3 presents the clothing pressure values for the two types of swimsuit materials and five body types (hourglass, apple, rectangle, triangle, and inverted triangle) and five swimming poses (overhead arm raise, forward bend, T-pose, flutter kick position, and cobra pose).

**Table 3:** Digital clothing pressures data collected by virtual try-on.

Body type		Swimming pose									
	Position	Digital clothing pressures (unit: kPa)									
		Overhead arm raise		Forward bend		T-Pose		Flutter kick position		Cobra pose	
Hourglass	Fabric^a^	A	B	A	B	A	B	A	B	A	B
	P1 (Chest)	33.78	33.78	31.07	31.07	32.41	32.41	35.78	35.78	36.44	36.44
	P2 (Back)	27.27	27.27	21.06	21.06	13.70	13.70	21.83	21.83	29.86	29.86
	P3 (Waist)	17.61	17.61	19.93	19.93	17.37	17.37	14.11	14.11	16.30	16.30
	P4 (Abdomen)	16.20	16.20	14.22	14.22	20.09	20.09	20.25	20.25	23.94	23.94
Apple	Fabric	A	B	A	B	A	B	A	B	A	B
	P1 (Chest)	24.84	24.84	25.98	25.98	28.64	28.64	30.64	30.64	27.67	27.67
	P2 (Back)	27.42	27.42	23.66	23.66	20.07	20.07	26.61	26.61	19.04	19.04
	P3 (Waist)	29.14	29.14	28.47	28.47	28.15	28.15	30.14	30.14	25.70	25.70
	P4 (Abdomen)	26.28	26.28	41.96	41.96	35.94	35.94	26.27	26.27	33.15	33.15
Rectangle	Fabric	A	B	A	B	A	B	A	B	A	B
	P1 (Chest)	21.82	21.82	18.73	18.73	25.08	25.08	24.10	24.10	22.26	22.26
	P2 (Back)	21.82	21.82	19.03	19.03	16.51	16.51	17.53	17.53	16.21	16.21
	P3 (Waist)	17.21	17.21	17.18	17.18	19.21	19.21	18.89	18.89	16.69	16.69
	P4 (Abdomen)	24.02	24.02	16.71	16.71	24.21	24.21	21.84	21.84	23.64	23.64
Triangle	Fabric	A	B	A	B	A	B	A	B	A	B
	P1 (Chest)	23.37	23.37	27.36	27.36	26.96	26.96	24.03	24.03	24.21	24.21
	P2 (Back)	16.01	16.01	26.02	26.02	10.86	10.86	19.23	19.23	15.68	15.68
	P3 (Waist)	12.51	12.51	14.59	14.59	12.23	12.23	14.37	14.37	14.40	14.40
	P4 (Abdomen)	16.80	16.80	38.12	38.12	12.68	12.68	14.44	14.44	27.41	27.41
Inverted Triangle	Fabric	A	B	A	B	A	B	A	B	A	B
	P1 (Chest)	33.13	33.13	29.50	29.50	33.72	33.72	30.86	30.86	40.76	40.76
	P2 (Back)	17.01	17.01	18.79	18.79	19.08	19.08	37.82	37.82	16.02	16.02
	P3 (Waist)	25.91	25.91	18.49	18.49	21.07	21.07	21.02	21.02	18.01	18.01
	P4 (Abdomen)	26.78	26.78	23.49	23.49	28.08	28.08	24.10	24.10	24.57	24.57

^a^Fabric A: Nylon 80% and spandex 20%; Fabric B: Polyester 80% and spandex 20%.

As illustrated in Table 3, CLO 3D cannot numerically differentiate clothing pressure differences between the two materials across all body parts (chest, back, waist, and abdomen) for all five body types and swimming poses. This is because CLO 3D generally includes only highly basic physical property information, including the stretch and recovery of a limited number of fabrics that are commonly known. Essentially, since CLO 3D does not have sufficient information on the various types of fibers and fabrics widely used in the industry and everyday wear, it fails to present the compressive force differences between nylon and polyester in this study. Moreover, the current CLO 3D basically considers the static state of the wearer when calculating clothing pressure; therefore, it fails to reflect dynamic deformation such as changes in fabric tension and friction as a result of wearer movement. Therefore, no surface pressure differences were observed based on the material for all poses despite applying five different swimming poses in this study.

**Fig 7:**
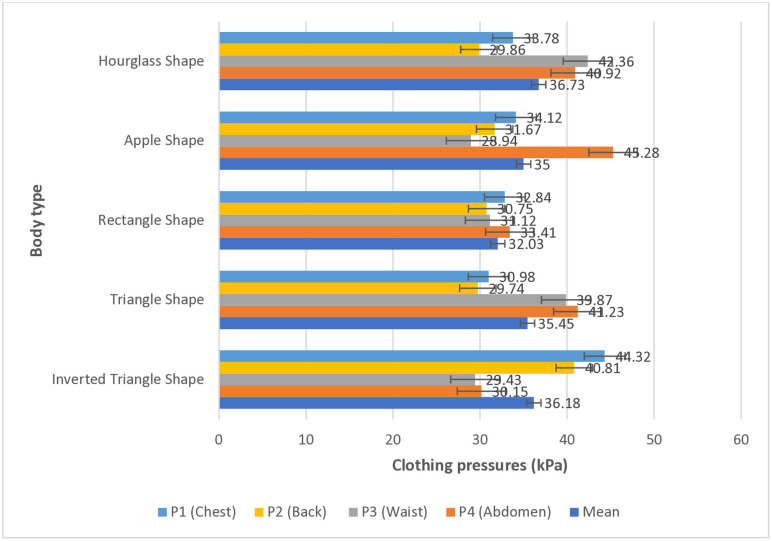
Numerical comparison of mean clothing pressure by body part according to body type (unit: kPa).

**Fig 8:**
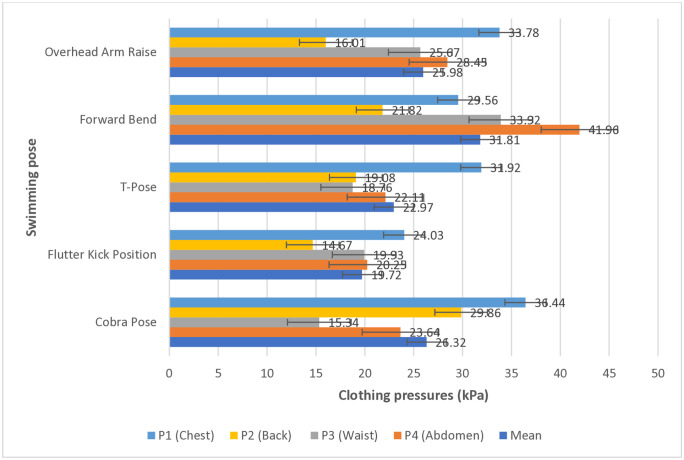
Numerical comparison of mean clothing pressure by body part according to swimming pose (unit: kPa).

Next, the study numerically compared the compressive force the swimsuits exerted on individual body parts according to body type (Fig 7). Regardless of the swimming pose, the body type with the highest mean clothing pressure across the entire body was the hourglass shape (36.73 kPa), whereas the body type with the lowest mean clothing pressure was the rectangle shape (32.03 kPa). Specifically, individuals with an hourglass figure may experience discomfort owing to increased overall pressure exerted by one-piece swimsuits during swimming involving various poses. Conversely, individuals with a rectangle shape can swim with relatively greater comfort, as the swimsuit exerts least pressure overall, regardless of the swimming pose. To verify the stability of the pressure data, error bars representing ± standard deviation (SD = 2.0 kPa) were added to Figs 7 and 8.

Specifically, individual garment pressure values for the four body parts (chest, back, waist, and abdomen) were compared and analyzed according to body type. For hourglass shape, characterized by larger chest and hips and well-defined waist, a relatively higher clothing pressure was observed on the waist (P3) and abdomen (P4), at 42.36 kPa and 40.92 kPa, respectively. This can be interpreted as the fabric tension being relatively higher, with pressure concentrating on specific body parts owing to the characteristic hourglass shape, where the waist circumference narrows sharply compared to the chest and hips. Meanwhile, a relatively lower surface pressure was observed on the chest (P1) and back (P2), at 33.78 kPa and 29.86 kPa, respectively, considering that these areas have gentle curves and larger surface areas, allowing for the distribution of pressure, resulting in lower pressure values.

For the apple shape, where the body fat is concentrated on the abdomen and waist, the highest clothing pressure (45.28 kPa) was observed on the abdomen (P4). Regardless of the type of swimsuit material, the abdomen was under strong pressure because of the large circumference. Contrary to expectations, the lowest garment pressure (28.94 kPa) was observed on the waist (P3), attributable to the pressure exerted, leading to the redistribution of pressure on the waist nearby.

In the rectangle shape, where there is not much difference in chest, waist, and hip widths, clothing pressure distribution across all body parts (chest 32.84 kPa; back 30.75 kPa; waist 31.12 kPa; and abdomen 33.41 kPa) was relatively uniform. This can be attributed to the lack of distinct body curves in this body type, resulting in a relatively even distribution of tension on the swimsuit fabric.

Meanwhile, for the triangle shape, where the lower body is more developed than the upper body, greater clothing pressure was observed on the abdomen (P4) and waist (P3), at 41.23 kPa and 39.87 kPa, respectively. This is attributable to the fabric being pulled toward the lower body, increasing fabric tension at the waist and abdomen. Conversely, relatively lower surface pressure was observed on the back (P2) and chest (P1), at 29.74 kPa and 30.98 kPa, respectively.

For the inverted-triangle shape, where the shoulders and chest are wider and waist and hips are narrower, clothing pressure increased on the chest (P1) and back (P2) contrary to the triangle shape, at 44.32 kPa and 40.81 kPa, respectively. This can be attributed to increased fabric tension on relevant body parts because this body type has a developed upper body, causing the fabric to be pulled upward. Meanwhile, low garment pressure was observed on the waist (P3) and abdomen (P4), at 29.43 kPa and 30.15 kPa, respectively, likely because of the generally narrower lower body, resulting in reduced compression on relevant body parts.

Various swimming poses led to different magnitudes and trends of surface pressure from swimsuits for different body parts. Fig 8 presents the mean compressive force values for individual body parts according to swimming poses regardless of body type. The pose with the highest mean garment pressure across the body is the forward bend (31.81 kPa), while the lowest mean clothing pressure is experienced in the flutter kick position (19.72 kPa). In essence, regardless of body type, the highest pressure is experienced during a forward-bend pose in a standard one-piece swimsuit, which could lead to discomfort. Contrastingly, in the flutter kick position, the wearer experiences the lowest pressure, which may result in a relatively higher comfort level.

Individual clothing pressure values for various body parts were compared and analyzed according to swimming pose. During the overhead arm raise pose, the highest garment pressure was observed on the chest (P1), at 33.78 kPa. Conversely, the lowest garment pressure was observed on the back (P2), at 16.01 kPa, suggesting that when the arms are raised, the fabric stretches over the protruding chest area, increasing pressure, whereas the relatively flat back area is more slack, reducing pressure.

In the forward bend position while preparing for diving, the highest clothing pressure was observed on the abdomen (P4), at 41.96 kPa, across all five swimming poses and four body parts. Given the nature of this movement, where the upper body bends forward, the curvature of the abdomen increases significantly. Consequently, both horizontal and vertical tension in the garment and fabric on the abdomen increase simultaneously, resulting in concentrated pressure on the abdomen. However, similar to the overhead arm raise, the lowest clothing pressure was observed on the back (P2), at 21.82 kPa during the forward bend.

In the T-pose, which involves shoulder and upper body stretching, similar moderate compressive force values were observed on the back (P2), waist (P3), and abdomen (P4), at 19.08 kPa, 18.76 kPa, and 22.11 kPa, respectively. However, a relatively higher clothing pressure of 31.92 kPa was observed on the chest (P1), although the value is not high compared to the clothing pressure observed on the abdomen (P4) during the forward bend pose (41.96 kPa) or the chest (P1) during the cobra pose (36.44 kPa), which will be discussed later. These results can be interpreted as follows: the T-pose involves raising the arms horizontally to the sides, leading to the swimsuit fabric stretching horizontally at the chest area, generating some pressure. However, the overall increase in tension remains limited, as there are no significant changes in body curvature. In contrast, the back, waist, and abdomen showed limited change in body shape during this pose, allowing the pressure to be evenly distributed.

In the flutter kick position, which represents the kicking posture in freestyle or backstroke, a relatively even and moderate distribution of garment pressure was observed across the body, including the chest (P1), compared to the other four poses (S.D: 4.71 kPa). This is likely because the position involves limited upper-body movement or curvature changes, maintaining a horizontal body posture, and allowing the tension applied to the garment to be relatively evenly distributed. Even the highest clothing pressure (24.03 kPa) observed on the chest (P1) was relatively lower compared with other positions. These findings suggest that individuals wearing a one-piece swimsuit experience overall reduced pressure and relatively higher comfort in this swimming pose.

In the cobra pose, where the upper body is arched backward and the waist and abdomen stretch, the lowest clothing pressure of 15.34 kPa was observed on the waist (P3), followed by the abdomen, at 23.64 kPa (P4). Meanwhile, the chest (P1) and back (P2) experienced higher clothing pressure, at 36.44 kPa and 29.86 kPa, respectively, because in this position, the waist and abdomen stretch like a bow, relaxing the fabric tension in those areas and reducing clothing pressure. In contrast, the chest and back curve along the body’s curvature, causing the fabric to stretch, with pressure concentrated in those areas.

Table 4 summarizes the results of a three-way ANOVA conducted to examine the effects of fabric type, body type, and swimming posture on clothing pressure in the CLO 3D simulation. The analysis revealed statistically significant main effects for all three independent variables: fabric type (*F* = 177.56, *p* < .001), body type (*F* = 30.79, *p* < .001), and posture (*F* = 93.18, *p* < .001). However, no statistically significant interaction effects were found among the three factors (all *p* > .05), suggesting that their influences on clothing pressure are independent and additive within this model.

**Table 4:** Three-way ANOVA results for effects of fabric, body type, and posture on clothing pressure.

Source	df	SS	MS	*F*	*P-value*
Fabric	1	140.94	140.94	177.563	<.001***
Body type	4	97.77	24.44	30.791	<.001***
Posture	2	147.93	73.97	93.183	<.001***
Fabric*Body Type	4	3.49	0.87	1.098	0.375
Fabric*Posture	2	4.09	2.04	2.574	0.093
Body Type*Posture	8	7.84	0.98	1.235	0.313
Fabric*Body Type*Posture	8	5.13	0.64	0.807	0.602
Residuals	30	23.81	0.79		

*p < .05, **p < .01, ***p < .001.

Considering that this study measured garment pressure in a virtual fitting state using CLO 3D, it was necessary to compare the findings with other studies that evaluated surface pressure based on actual human-wear trials. Accordingly, the body parts exhibiting the highest and lowest clothing pressure in previous studies that examined sportswear were analyzed (Table 5). Previous studies have suggested that clothing pressure distribution of various sportswear are closely related to movements and functions of the relevant sport. This study aimed to analyze how compressive force distribution of various types of sportswear differs from that of swimsuits targeted in this study.

**Table 5:** Comparison of clothing pressure across different sportswear types.

Sportswear type	Highest clothing pressure areas	Lowest clothing pressure areas	Functional characteristics	Related research
Compression sportswear	Chest, thighs, calves	Waist	Muscle recovery, improved circulation, posture stabilization	Liu, et al. [28]
Corset-type harness	Waist (load-bearing areas)	–	Load distribution, safety enhancement	Kwon, et al. [30]
Yoga wear	Waist, thighs	Chest, back	Stretchability, breathability, minimizing restriction of movement	Teyeme, et al. [31]
Cycling shirt	Chest, waist	back	Aerodynamic fit, moisture management	Liu, et al. [32]
Medical compression-wear	Abdomen, thighs	Back, arms	Posture correction, pressure therapy, circulation improvement	Brubacher et al. [33]
Senior women’s activewear	Chest, upper back	Waist, arms	Physical activity support, ergonomic fit	Choi, et al. [34]
Pressure injury prevention clothing	Hips, Buttocks	Waist, thighs	Injury prevention, pressure relief, comfortable wearability	Salgueiro-Oliveira, et al. [35]

The present study aligns with previous research indicating that the mean clothing pressure on various body parts is strategically designed based on the purpose of wearing sportswear. The garment pressure applied to the body tended to concentrate on areas where performance improvements, including strength, are required for body support and movement execution. However, areas that require flexibility and comfort for movement tended to show relatively lower pressure. The findings suggest that when designing functional clothing, including swimsuits, adjusting the pressure on different body parts critically influences a product’s functionality and wearability. Compression sportswear generates greater surface pressure on the chest, thighs, and calves (the generally higher pressure on the chest in swimsuits aligns with the findings of this study) and helps promote blood circulation and muscle recovery [28]. Corset-type harnesses exert concentrated pressure on the waist, distribute the load, and enhance stability [30]. Yoga wear generates greater pressure on the waist and thighs with a design that considers stretchability and breathability to minimize movement restriction [31]. A cycling shirt applies higher pressure on the chest and waist for an aerodynamic fit while exhibiting relatively lower pressure on the back [32].

Medical compression-wear generates greater pressure on the abdomen and thighs, contributing to posture correction and circulation improvement through pressure therapy [33]. Senior women’s active wear supports physical activity by applying higher pressure on the chest and upper back, reflecting an ergonomic fit [34]. Clothing aimed at preventing pressure injury creates higher pressure on the hips and buttocks, protecting the body and relieving pressure [35].

The functionality of sportswear reported in previous studies [20,28,30–35], although it may vary depending on the type of sportswear/functionality wear, generally provides higher clothing pressure on areas related to support or performance improvement (e.g., chest, thigh, abdomen). In contrast, areas around joints that require ease of movement or sensitive areas that need caution to prevent injury (e.g., arms, back, waist) tended to experience relatively lower pressure in general. This tendency aligns with the clothing pressure results for swimsuits according to movement, as analyzed in this study.

### 3.3. Comparison between CLO 3D simulation and Mannequin-based wear trial

In this study, the clothing pressure values obtained from CLO 3D simulations and mannequin-based wear trials were compared using the hourglass body type to evaluate the extent to which virtual simulations align with physical measurements. As shown in Table 6, the pressure values measured from the mannequin-based trial were consistently higher than those generated by CLO 3D across all body regions (P1–P4). The chest region (P1) exhibited the largest discrepancy, with an average difference exceeding 9 kPa between the two methods. The abdomen (P4) and waist (P3) regions also showed noticeable differences, with mannequin-based values approximately 7–8 kPa higher than those from CLO 3D. Only the back (P2) region exhibited a relatively small gap between the two methods.

**Table 6:** Mannequin wear trial pressure data.

Body type		Swimming pose					
	Position	Clothing pressures (unit: kPa)					
		Overhead arm raise		Forward bend		T-Pose	
Hourglass	Fabric^a^	A	B	A	B	A	B
	P1 (Chest)	44.10	46.20	40.20	42.50	41.00	42.80
	P2 (Back)	32.60	33.90	27.40	28.60	19.80	20.10
	P3 (Waist)	23.70	24.40	26.10	27.30	23.30	24.50
	P4 (Abdomen)	21.30	22.20	19.70	20.80	26.80	28.00

^a^Fabric A: Nylon 80% and spandex 20%; Fabric B: Polyester 80% and spandex 20%.

The repeated-measures ANOVA results presented in Table 7 confirmed that the measurement method (CLO 3D vs. mannequin-based trial) had a statistically significant effect on clothing pressure values across all body regions. The chest region (P1) showed particularly high significance (*F* = 1198.30, *p* < .001), while the waist (P3) and abdomen (P4) regions also showed significant differences (*F* = 417.20 and *F* = 118.55, respectively; both *p* < .001). The back region (P2), although with a smaller discrepancy, still showed statistical significance (*F* = 58.70, *p* = 0.0015).

**Table 7:** Repeated-measures ANOVA results comparing CLO 3D Mannequin (hourglass body type).

Body region	Dependent variable: Clothing pressure				
	Sum of squares	*d.f.*	Mean square	*F*	*p-value*
Chest (P1)	482.52	1	482.52	1198.30	<.001***
Back (P2)	21.34	1	21.34	58.70	0.0015**
Waist (P3)	163.82	1	163.82	417.20	<.001***
Abdomen (P4)	44.18	1	44.18	118.55	0.0006***

*p < .05, **p < .01, ***p < .001.

**Fig 9:**
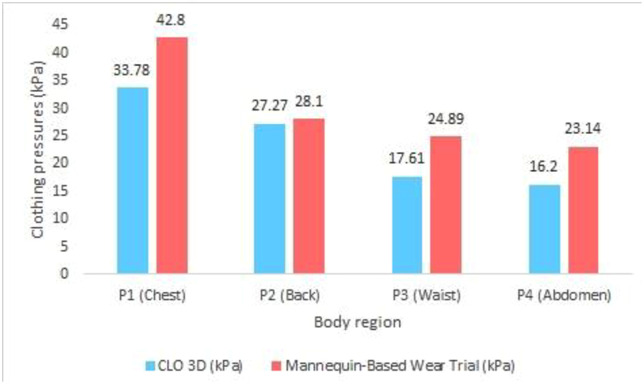
Comparison of clothing pressure values by body region between CLO 3D simulation and Mannequin (hourglass body type).

These findings suggest that while CLO 3D can reasonably reflect relative pressure distribution patterns across different postures and body parts (Fig 9), it tends to underestimate absolute pressure values when compared to physical trials. Therefore, mannequin-based testing may serve as a valuable complement to simulation-based garment evaluations, especially for applications requiring precise pressure assessment.

### 3.4. Suggestions to optimize CLO 3D functions

CLO 3D is used in both the fashion industry and academia for various purposes, including digital clothing simulation, virtual fitting, and testing the physical properties of fabrics. Its use is gradually transcending beyond the fields of clothing, including general fashion apparel, sportswear, and medical wear, to include the creation of avatar garments in games and metaverse. Accordingly, it offers advantages, such as reducing the cost of producing actual physical samples, enabling eco-friendly production, and allowing quick design modifications. However, the current CLO 3D system has a few functional and technical limitations in analyzing clothing pressure. Therefore, research that identifies these limitations and suggests measures for improvement holds significant importance for the advancement of the fashion industry and digital fashion technology.

With a focus on swimsuits, as observed in this study, the current CLO 3D system presents four major limitations in analyzing clothing pressure. Although this study used static poses to represent key swimming-related positions, actual swimming involves continuous and dynamic movements. The current CLO 3D system does not support motion-based simulation, and therefore cannot fully reflect the time-varying deformation and pressure changes that occur during real swimming strokes. This limitation should be considered when interpreting the results, as static simulation may underestimate or misrepresent garment pressure in dynamic conditions. Future research may benefit from integrating animation-based or motion-capture-enhanced simulation environments to better replicate dynamic pressure behavior.

**Fig 10:**
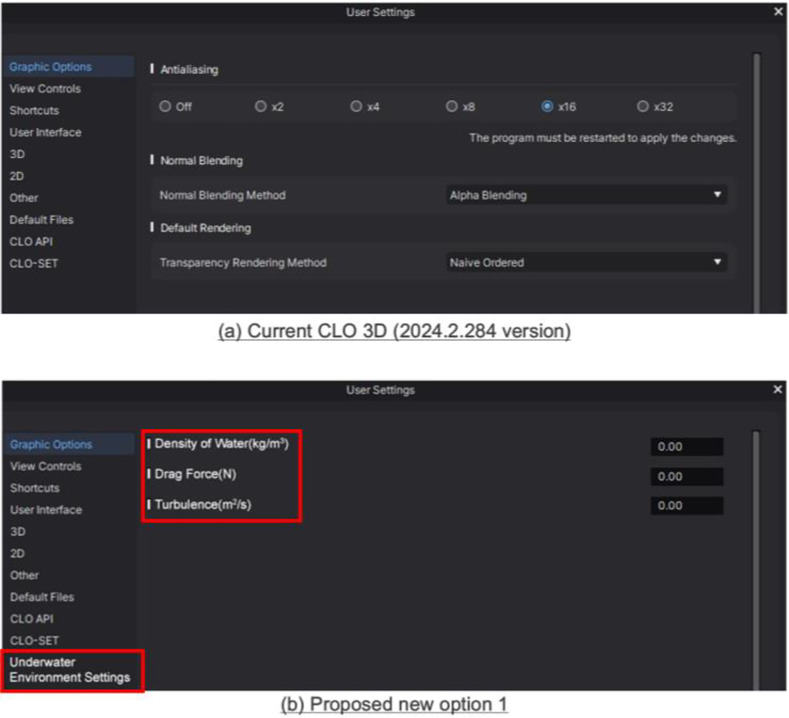
Current CLO 3D (2024.2.214 version) and proposed option 1: Setting the underwater environment.

First, it performs simulations in a standard dry environment and does not reflect the underwater environment. Factors such as water pressure, buoyancy, and underwater drag force affect compressive force not only during swimming movements but also in a static state. Accordingly, this study proposes an underwater simulation module for CLO 3D, alongside an underwater environment setting function for the module that allows software users to predict underwater swimsuit fabric deformation in real-time by entering the density of water (kg/m3), drag force (N), and turbulence (m2/s) (Fig 10). This would facilitate precise clothing pressure analysis reflecting an actual underwater environment, rather than relying solely on existing air-based simulations.

**Fig 11:**
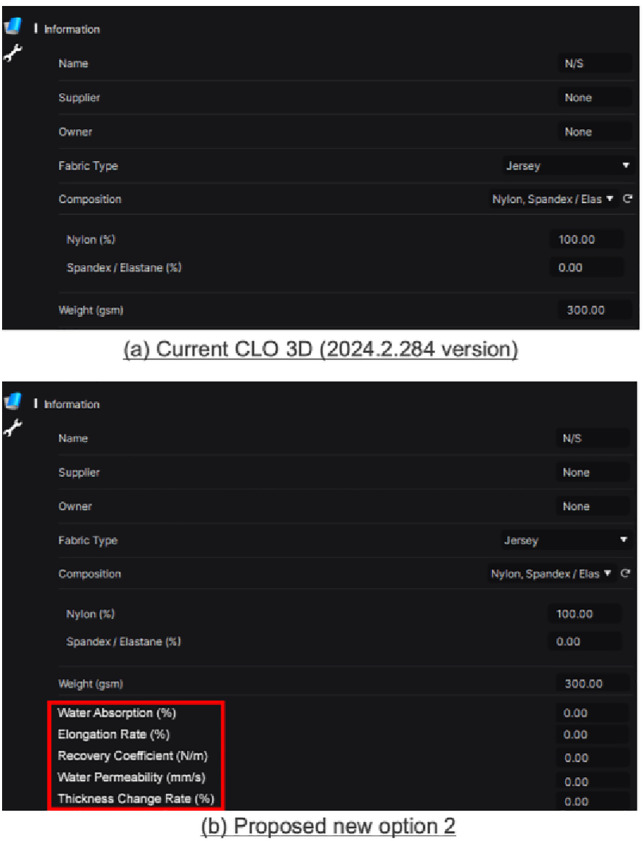
Current CLO 3D (2024.2.214 version) and proposed option 2: Providing a database of swimsuit fabric properties in a wet state.

Second, it does not sufficiently reflect the changeable physical properties of fabrics. The physics engine provided in the current system only considers basic fabric types (jersey, woven, denim, leather, rib, chiffon, satin, corduroy, velvet, lace, mesh, tulle, knitted, wool, and fleece), composition, and weight to predict the approximate appearance and fit of garments in a dry state. It does not reflect the properties of swimsuit fabrics, such as spandex, nylon, polyester, etc. in a wet state. Textile fibers generally exhibit significantly different properties, especially strength and elongation, in dry and wet states [36]. For example, as reported in prior studies [36], nylon may exhibit up to 20% increased elongation in wet conditions, which can significantly impact pressure levels during wear. This limitation represents a fundamental bottleneck in applying CLO 3D to simulate aquatic garments such as swimsuits. Therefore, this study recommends a database of properties of commercially-available swimsuit fabrics in a wet state within the software library, specifically water absorption rate (%), elongation rate (%), recovery coefficient (N/m), water permeability (mm/s), and thickness change rate (%) (Fig 11). Accordingly, software users would be able to distinguish between the dry and wet states of the fabric they intend to assess and more accurately predict appearance, fit, and clothing pressure, as well as wearer comfort in a wet state. Furthermore, if necessary, they can manually enter the aforementioned properties under wet environmental conditions.

Moreover, the lack of pressure differences observed between nylon and polyester fabrics (Table 3) may be partly attributed to the absence of wet-state material parameters in the CLO 3D library. Prior studies have reported that nylon exhibits up to 20% increased elongation under wet conditions [34], which would significantly reduce the pressure during actual use. This highlights the critical need to incorporate humidity-dependent material behavior into CLO 3D to ensure accurate pressure prediction and simulation fidelity for aquatic garments. In this study, fabric types were selected from the CLO 3D default material library, with nylon and polyester both implemented using standard software presets. These presets do not offer editable options for wet-state properties such as elongation, water absorption, or recovery behavior. Consequently, despite selecting two different materials, the simulation yielded no significant pressure differences (Table 3), which may be attributed to the lack of distinct mechanical behavior settings in the current CLO 3D system. This limitation underscores the need for customizable wet-state material input to improve the accuracy of aquatic garment simulations.

**Fig 12:**
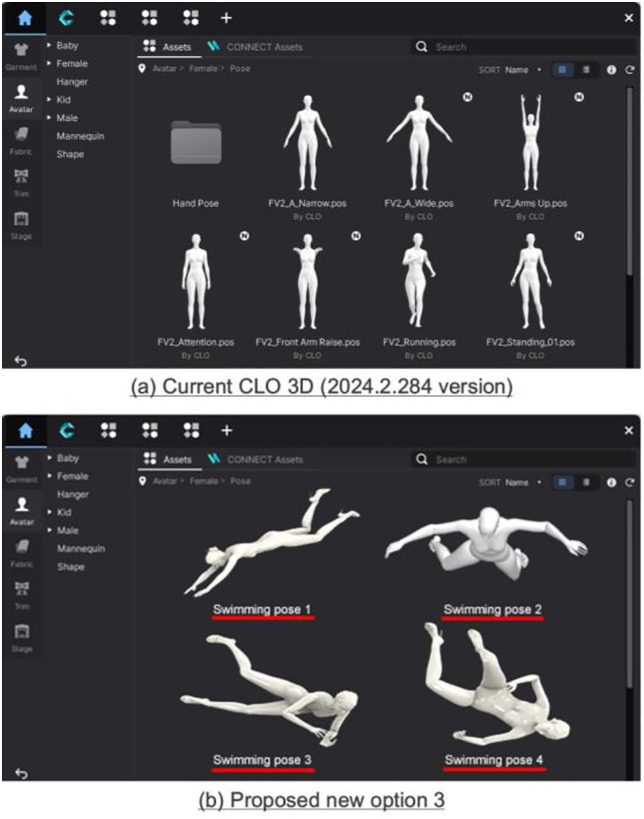
Current CLO 3D (2024.2.214 version) and proposed option 3: Adding movements.

Third, it predicts clothing pressure only in the static state, thereby failing to analyze garment changes during movement, including swimming. For example, during swimming motions such as kicks, strokes, and turns, the swimsuit is pulled as the body stretches, changing the fabric pressure exerted on the wearer, which the current CLO 3D cannot simulate with precision. Accordingly, this study proposes a function that allows users to select between static and dynamic states of the wearer and add movements for analysis in the dynamic state (Fig 12). A motion simulation function can be included to predict changes in garment pressure when a specific movement is repeated, enabling the development of an animation-based simulation system that reflects the wearer’s dynamic state.

**Fig 13:**
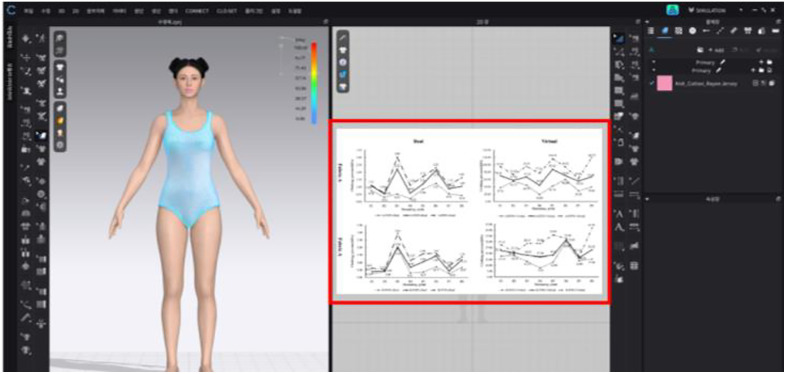
Proposed option 4: Automatically correcting differences between measured clothing pressure data and CLO 3D data.

Finally, clothing pressure data based on virtual simulation of CLO 3D may differ from surface pressure measurements based on the use of an actual pressure sensor and human-wear trials. It is challenging to achieve an extremely precise representation of actual clothing pressure on the software given the aforementioned limitations of the current CLO 3D, which persist despite advancements in virtual simulation technology. Accordingly, this study proposes a function that enables comparison and calibration of actual garment pressure measurement data—obtained through pressure sensor and human-wear trials—with the simulated clothing pressure data of CLO 3D (Fig 13). This automatically corrects the difference between actual measurement data (database obtained from existing studies) and CLO 3D data using AI machine learning, thereby increasing the accuracy of CLO 3D clothing pressure simulations.

The four proposed functions of CLO 3D will help further develop digital fashion technology in the era of the Fourth Industrial Revolution, while improving the practical skills of designers in developing functional clothing. These functions enable designers to enhance both efficiency and accuracy by providing precise simulations for developing various sportswear, including swimsuits, while also allowing consumers to obtain detailed information about the comfort of specific areas of the product during movement, which is typically difficult to experience before making a purchase.

## 4. Conclusion

This study qualitatively and quantitatively analyzed the clothing pressure of adult women’s full-body swimsuits based on various female body types and swimming movements using the CLO 3D virtual fitting system. Specifically, five body types (hourglass, apple, rectangle, triangle, and inverted triangle) and five movements (overhead arm raise, forward bend, T-pose, flutter kick position, and cobra pose) were selected, and garment pressure on the chest, back, waist, and abdomen was measured by applying nylon (80% nylon + 20% spandex) and polyester (80% polyester + 20% spandex). Also, based on the limitations of the current CLO 3D detected, this study proposed functions such as setting the underwater environment, providing a database of swimsuit fabric properties in a wet state, adding movements, and automatically correcting differences between measured compressive force and CLO 3D data. Furthermore, to verify the reliability of the CLO 3D output, a supplementary mannequin-based wear trial using a pressure sensor system was conducted. While CLO 3D tended to underestimate absolute pressure values, it successfully reflected the relative distribution patterns observed in the physical measurements.

This study is the first to analyze clothing pressure in swimsuits under various body types, movements, and fabric conditions using CLO 3D. It is expected to make a practical contribution to the fashion and sportswear industries as well as academic research. Specifically, from an industrial perspective, the findings can provide data-driven design directions to enhance wearability and functionality in developing swimsuits, and can even serve as foundational data to help CLO 3D evolve into an advanced digital fashion simulation technology with high practicality and precision. Furthermore, from an academic perspective, this study holds significance by demonstrating the potential for CLO 3D-based sportswear research without relying on traditional human-wear trials, by validating simulations through quantitative mannequin-based pressure measurements. However, this study was limited to women’s one-piece swimsuits and female adult body types. Therefore, future research should include different types of swimsuits (e.g., two-piece, swim jammer, racing suit) and different wearers (male adults, children, and the elderly).

## Supporting information

S1 Table Raw numerical data of clothing pressure (kPa) obtained from CLO 3D simulations across body types and swimming poses. (XLSX)

S2 Table Raw numerical data of clothing pressure (kPa) measured in the mannequin-based wear trial. (XLSX)

S3 Table Input fabric property parameters used for CLO 3D simulation. (XLSX)

S4 Table Physical properties of actual swimsuit fabrics used in the mannequin-based evaluation. (XLSX)
